# Household Hints and Recipes

**Published:** 1881-07

**Authors:** 


### Household Hints & Recipes

TO SILVER NON-METALLIC SUBSTANCES, apply alternately solutions of gallic or pyrogallic acid and of nitrate of silver, the latter containing ammonia, but not in excess. Textile fabrics are run through one of hyposulphite of soda.

---

TO DARKEN OAK FRAMING.—Take one ounce of carbonate of soda, and dissolve in half-pint boiling water; take a sponge or piece of clean rag, saturate it in the solution, and pass gently over the wood to be darkened, so that it is wet evenly all over; let it dry for 24 hours. Try first on an odd piece of wood to see color; if too dark, make the solution weaker by adding more water; if not dark enough, give another coat. This may always be kept ready for use in a corked bottle.

---

ASHES AS A SUBSTITUTE FOR EMERY.—A manufacturer whose business requires the use of large amounts of emery, has been trying an experiment with the ashes of anthracite coal, and he affirms that he has obtained good results from the use of ashes as a substitute for the finer grades of emery. He takes ashes and saturates them with water, the liquid being poured off after standing an hour or two, then being poured off again, and so until he obtains several grades, down to a substitute for emery flour. When dried, the deposit cuts readily and leaves a satisfactory surface.

---

TO TRANSFER FIGURES, NAMES, PICTURES, ETC., UPON GLASS, the following method of Leceve, of Paris, may be employed: Glass which is thinly silvered is coated with a very thin coat of asphalt. This is done by dissolving Syrian asphalt, such as is sold by photographic dealers, in benzine, and coating the glass with the solution, without exposure to direct sunlight. A photographic cliche, or a properly cut pattern of dark paper, pasteboard, etc., is laid upon the asphalt coat when dry, and the whole then exposed to the rays of the sun, which will render the asphalt, wherever the latter is exposed, insoluble. The protected asphalt coating is then washed away with benzine and the "silver" coating below it with nitric acid, while the drawing or pattern will appear in silvered lines and figures upon the glass.—*Industrie-Blatter.*

TO ANNEAL LAMP CHIMNEYS.—The following receipt is from a Leipsic journal, devoted to the glass interest: Place your tumblers, chimneys or vessels which you desire to keep from cracking, in a pot filled with cold water; add a little cooking salt; allow the mixture to boil well over a fire, and then cool slowly. Glass treated in this way is said not to crack even if exposed to very sudden changes of temperature. Chimneys are said to become very durable by this process, which may also be extended to crockery, stoneware, porcelain, etc. The process is simply one of annealing, and the slower the process, especially the cooling portion of it, the more effective will be the work.

---

CEMENT FOR RUBBER.—Powdered shellac is softened in ten times its weight of strong water of ammonia, whereby a transparent mass is obtained, which becomes fluid after keeping some little time without the use of hot water. In three or four weeks the mixture is perfectly liquid, and, when applied, it will be found to soften the rubber. As soon as the ammonia evaporates the rubber hardens again—it is said quite firmly—and thus becomes impervious both to gases and to liquids. For cementing sheet rubber or rubber material in any shape, to metal, glass, and other smooth surfaces, the cement is highly recommended.

---

LIQUID GLUE.—A glue ready for use is made by adding to any quantity of glue common whiskey instead of water. Put both together in a bottle, cork it tight, and set it for three or four days, when it will be fit for use without the application of heat. Glue thus prepared will keep for years, and is at all times fit for use except in very cold weather, when it should be set in warm water before using. To obviate the difficulty of the stopper getting tight by the glue drying in the mouth of the vessel, use a tin vessel with the cover fitting tight on the outside to prevent the escape of the spirit by evaporization. A strong solution of isinglass made in the same manner is an excellent cement for leather.

---

SALICYLIC ACID FOR BEE-STINGS.—Although salicylic acid, from having been too highly extolled, has fallen somewhat into disfavor, there can be no doubt that it is useful in the case of bee-stings. An Austrian paper recommends the following treatment: First, to remove the sting as quickly as possible with a forceps or by scratching with a finger, but never between the thumb and forefinger, because this squeezes more of the poison into the wound. Next squeeze the wound until a drop of blood comes out, and rub the place, as large as a dollar, with an aqueous or dilute solution of salicylic acid into the wound with the hypodermic syringe. After this the spot is painted with collodion to keep out the air. A sting treated thus causes little or no pain, slight inflammation and swelling, and is not followed by nettle fever or lameness in the most sensitive and nervous individuals.—*Scientific American.*

---

DRINKS FOR FEVER PATIENTS.—Drinks made from fresh or preserved fruits are sometimes useful in fevers. Rhubarb tea is a very refreshing spring beverage. Slice about two pounds of garden rhubarb, and boil for a quarter of an hour in a quart of water; strain the liquor into a jug, adding a small quantity of lemon peel, and some sugar to taste; when cold it is fit for use. Apple water may be made in the same manner. The apples should be peeled and cored. Sugar should not be added to either of the above until after the liquor is removed from the fire. In the absence of fresh fruit, a pleasant beverage may be prepared by stirring sufficient raspberry jam or current jelly into the required quantity of water, straining the liquor before giving it to the patient.

---

GLUCOSE.—The manufacture of glucose has become so large and important a business that it would be utterly beyond the limits which we could afford, to give a complete description of the processes by which it is manufactured at present. Immense quantities are made in this country from Indian corn, and are put upon the market either a solid form (granular or in lumps) or in form of a syrup, which is largely used as a substitute or adulterant of true sugar-house syrup and also of honey. In general, the process of manufacture may be thus described. 400 part of water are mixed with 4 parts of sulphuric acid, and the mixture raised to boiling. Meanwhile 100 parts of starch (or potato flour, or corn flour, etc.) are made into a milk with water, then added to the boiling dilute acid, under constant agitation, and the whole boiled until the dextrin, which is formed at first, is completely converted into glucose, which may be recognized by the fact that 1 part of the liquid is no longer precipitated by 6 parts of absolute alcohol. Of course, a slight precipitate always occurs, which is owing to the presence of substances, chiefly derived from the starchy material, which are insoluble. The acid liquid is now mixed with lime, in whatever form it can be obtained cheapest (caustic lime, chalk, marble, etc.), the insoluble sulphate of calcium allowed to deposit, and the liquid decanted. This is now evaporated to a density of 15 or 16° Beaume, filtered through bone-black and further concentrated in vacuum-pans either to a syrup or to a point of crystalization. If the highly concentrated mass is allowed to completely solidify to a crystaline mass, the crystals turn out to be very impure. To produce colorless or white glucose, it is necessary to arrest the concentration at a certain point, and to separate the still syrupy portion of the mass from the crystals already formed. 100 parts of starch yield about 100 parts of sugar or 150 of syrup.

---

WORCESTERSHIRE SAUCE.—The genuine sauce has often been "imitated," yet though a few of the imitations approach it quite closely, none of them has ever equalled it in all respects. We do not know the composition of the original sauce, nor that of its imitations, and can give you only what we find quoted as the "reputed" formula :

| | | |
|---|---|---|
| Walnut catsup | 10 | gall. |
| Mushroom catsup | 10 | " |
| Madeira wine | 5 | " |
| White vinegar | 15 | " |
| Canton soy | 4 | " |
| Common salt | 25 | lbs. |
| Chillies, ground | 2 | " |
| Allspices powd | 1 | " |
| Coriander powd | 1 | " |
| Cloves | ½ | " |
| Mace | ½ | " |
| Cinnamon | ½ | " |
| Asafœtida | 2 | oz. |
| Brandy | 1 | gall. |

Macerate the asafœtida with the brandy until its soluble parts are extracted. Mix the catsups, wine, vinegar and salt, heat them to boiling, then add the spices and brandy, cover well and let cool. Boil 20 lbs. of hog's liver for 12 hours with 16 gallons of water, renewing the water from time to time. Then take out the liver, chop it fine, mix it with water, work it through a sieve, and mix the pulp with the sauce.

